# Fast-track- recovery surgery with a whey-protein-infused carbohydrate-loading drink pre-operatively and early oral feeding post-operatively among surgical gynaecological cancer patients: study protocol of an open-labelled, randomised controlled trial

**DOI:** 10.1186/s13063-020-04462-4

**Published:** 2020-06-16

**Authors:** Chiou Yi Ho, Zuriati Ibrahim, Zalina Abu Zaid, Zulfitri ‘Azuan Mat Daud, Nor Baizura Md Yusop

**Affiliations:** 1 Department of Nutrition and Dietetics, Faculty of Medicine and Health Science, Universiti Putra Malaysia, 43400 Serdang, Selangor Malaysia; 2 Dietetics and Food Service Department, National Cancer Institute, Ministry of Health, Jalan P7, Precint 7, 62250 Putrajaya, Malaysia

**Keywords:** Whey-protein carbohydrate-loading, Early oral feeding, Surgical gynaecological cancer

## Abstract

**Introduction:**

There has been growing evidence on the favourable outcomes of fast-track-recovery (FTR) surgery; to expedite recovery, minimise complications, and reduce the length of hospital stay for surgical patients. However, there is lack of evidence on the effectiveness of FTR in surgical gynaecological cancer (GC) patients. Most of the previous studies did not focus on feeding composition in the FTR surgery protocol. This study aims to determine the effectiveness of FTR feeding with a whey-protein-infused carbohydrate-loading drink pre-operatively and early oral feeding post-operatively on post-operative outcomes among surgical GC patients.

**Methods/design:**

This open-labelled, randomised controlled trial (RCT) will randomly allocate patients into intervention and control groups. Ambulated Malaysian aged over 18 years and scheduled for elective surgery for (suspected) GC, will be included in this study. The intervention group will be given whey-protein-infused carbohydrate-loading drinks on the evening before their operation and 3 h before their operation as well as started on early oral feeding 4 h post-operatively. The control group will be fasted overnight pre-operation and only allowed plain water, and return to a normal diet is allowed when bowel sounds return post-operatively. The primary outcomes of study are length of post-operative hospital stay, length of clear-fluid tolerance, solid-food tolerance and bowel function. Additional outcome measures are changes in nutritional status, biochemical profile and functional status**.** Data will be analysed on an intention-to-treat basis.

**Trial registration:**

ClinicalTrials.gov, ID: NCT03667755. Retrospectively registered on 12 September 2018;

Protocol version**: v**ersion 3 dated 27 September 2017.

## Strength and limitations of study

- This is an open-labelled, randomised controlled trial (RCT) study
- There is a large number of participants (*n* = 110 patients)
- This study is among the first few RCTs focussed on FTR surgery with pre-operative whey-protein-infused carbohydrate (CHO)-loading and post-operative early oral feeding on GC patients in the Southeast Asian region
- The limitation is that the post-operative observation is short, so the long-term effects of perioperative FTR surgery feeding with whey-protein-infused CHO-loading on wound-healing could not be assessed
- This is a single-centre study and the protocol used might not be applicable to other hospitals

## Background and rationale

Fast-track-recovery surgery (FTR) (also known as Enhanced Recovery After Surgery (ERAS)) has been proposed and established since 1999 by Professor Dr. Henrik Kehlet [1]. FTR is developed based on the concept of multimodal post-operative recovery programmes after it was realised that the unimodal intervention was impractical to solve multimodal perioperative morbidity problems. FTR surgery, also known as ERAS programmes, are increasingly been practiced in colorectal surgery [2], gastrointestinal surgery) [3] and various other surgical fields [4–6]. One of the crucial parts in the FTR programme is early perioperative feeding which is safe and beneficial in post-operative recovery [7]. Many studies have highlighted the general FTR programme which includes providing patients solely with carbohydrate (CHO)-loading pre-operatively and allowing early oral feeding with clear fluid and followed by solid food only after 500 mL clear fluid is tolerated post-operatively [7–10]. However, the effectiveness of providing a whey-protein-infused CHO-loading drink as the pre-operative CHO-loading drink remains unclear. Practical consideration for FTR surgery development in GC surgical patients is growing [11]. “The FTR programme includes evidence-based items designed to reduce perioperative stress, maintain post-operative physical function and accelerate recovery after surgery” [12]. Using a multi-modal stress-minimising approach has been shown repeatedly to reduce rates of morbidity, improve recovery and shorten length of hospital stay (LOS) after major colorectal surgery [13]. An updated consensus review of perioperative care of colorectal cancer patients has highlighted that the multimodal metabolic stress-minimising approach was demonstrated repeatedly to shorten LOS stay, promote recovery and reduce the morbidity rate after major colorectal surgery [14]. Clinical care of patients undergoing GC surgery is different between hospitals and countries; thus, it is necessary to understand the effectiveness of FTR surgery perioperative feeding specifically in Malaysia.

The primary modalities of cancer treatment are surgery, chemotherapy and radiotherapy; these may be used alone or in combination [15]. For those cancers which are well-defined and operable, surgery is the first-line treatment for GC [16]. Surgery, like an injury, causes catabolism which involves skeletal-muscle-tissue breakdown to release amino acids for wound-healing. Optimal nutritional status perioperatively supports speedy wound-healing, improves immunity and ensures the better post-surgical outcome. Other than calories and CHO, protein is crucial for post-operative recovery, promotes anabolism, slows down muscle catabolism and decreases the inflammatory phase [17]. Milk proteins are divided into two classes: whey and casein. Whey protein is a high-quality protein that is more easily digested and stimulates muscle-protein synthesis more than does casein. Fast digestion and absorption of whey protein is due to the high proportion of branched-chain amino-acid content which directly stimulates the early cellular processes involved in protein synthesis called initiation translation [18]. However, this might not be beneficial to people with milk allergy as they might also be allergic to whey protein [19].

Conventional pre- and post-operative feeding strategies, whereby prolonged fasting or rest for both the patient and their gastrointestinal tract, will delay patient recovery since the organic response to surgical trauma is enhanced by a prolonged period of fasting. Inadequate oral intake due to delayed oral feeding caused depletion of nutrient storage in a patient’s body. This is because of utilisation of energy which is converted from a protein source of body (muscle) which promotes catabolism. Thus, patients experience weight loss and muscle-mass loss post-operatively [20].

In order to overcome the complications of conventional surgical approaches, Kehlet and Wilmore [7] developed a multimodal perioperative protocol, named Enhanced Recovery after Surgery or the fast-track-recovery (FTR) surgery programme. One of keys to FTR programmes is a metabolic strategy to reduce perioperative stress and improve surgical outcomes. Nutritional intervention post-operatively is crucial. Studies of the FTR programme in gynaecological surgery has shown that FTR significantly reduce LOS and consequently leads to positive economic benefits without increasing readmission and complication rates [8, 9, 21]. Studies of the FTR protocol in gynaecological surgery have shown that FTR with pre-operative CHO-loading and post-operative early oral feeding significantly reduces LOS and consequently has positive economic benefits without increasing readmission and complication rates. The FTR protocol focus primarily on reducing perioperative stress, achieving satisfactory pain control, resumption of normal gastrointestinal function and early mobilisation [17, 20, 22].

While early oral feeding is the preferred mode of nutrition, the avoidance of any nutritional support therapy bears the risk of underfeeding during the post-operative course after major surgery. To simplify pre-operative fasting, beverages containing CHOs have been used and recommended in the FTR protocol. Carbohydrate-loading with solely a CHO drink was widely used and proven positive and with beneficial outcomes [17, 20, 22]. Whey protein contains essential amino acids, especially branch-chain amino acids, which have a high degree of digestibility and rapid absorption in the small bowel, which is rapidly used by skeletal muscle during stress and greatly stimulates protein synthesis [23]. Perrone et al. [5] concluded that whey protein infused with CHO drinks as the CHO-loading drink prior to operation improves post-operative muscle strength, reduces fatigue, anxiety and discomfort as well as lowers the endocrine-metabolic response to trauma. The study aims to determine the effectiveness of FTR surgery with a whey-protein-infused CHO-loading drink and early oral feeding among surgical GC patients.

## Methods/design

This study is an open-labelled, randomised controlled trial (RCT) conducted at the National Cancer Institute, Putrajaya, Malaysia. Since this is an open-labelled RCT study, blinding will not occur. The study is conducted in the Surgical Gynaecological Department which involves a multidisciplinary clinic and a female surgical ward. The RCT will conform to the Consolidated Standards of Reporting Trials (CONSORT) Statement for reporting RCTs with two arms, comparing an intervention group to a control group [24]. After consenting to participate, participants will be randomly allocating into the intervention group or the control group. Randomisation will be done using computer-generated random numbers.

### Participants

One hundred and ten participants fulfilling the eligibility criteria will be enrolled into the study. Ambulatory Malaysian women aged over 18 years and scheduled for elective surgery for (suspected) GC, will be included in this study. Those who are allergic to soy or whey protein, diagnosed with chronic renal disease, ischaemic heart disease or diabetic mellitus, or who are involved in other interventional studies are excluded. The time of enrolment will start from the admission day until the day of discharge (throughout hospitalisation for elective surgery).

### Recruitment procedure

Patients’ perioperative feeding will be managed according to the FTR surgery programme [1]. Candidates who have been referred to the Surgical Gynaecological Department and complying with the inclusion criteria will be approached to participate in the RCT study. Oral and written information about the study procedures will be provided by a gynaecological surgeon or a dietitian in a private consultation room. The gynaecological surgeons and dietitians involved in recruitment have been trained and instructed in the recruitment procedure in order to maximise the recruitment rate. Patients are provided with a patient information sheet and a study consent form and given sufficient time to consider and discuss participation with family members before decision-making. The dietitian will contact the patient to confirm participation in the study. A written informed consent form will be collected on the admission day. Participants will be required to attend the diet clinic to have an anthropometry and dietary assessment on the day of admission.

### Randomisation

Consented participants will be randomised into two groups: the intervention (CHO-P) group and the control group (CO), before baseline assessment. Randomisation is done by a computer-generated randomisation number which is prepared by an independent statistician. Randomised allocation numbers will be concealed in sealed envelopes by the study coordinator. The envelop will only be opened after consented and before baseline assessment.

### Intervention group (CHO-P)

The intervention group will receive a specific drink of a lactose-free, clear, tea-coloured, fruit-flavoured fluid containing 14% whey protein, 86% CHOs and 0% lipids. Participants in the CHO-P group will be given a 474-mL evening drink which provides 500 kcal and 18 g whey protein on the evening of the day before their operation. Three hours prior to their operation, participants will be provided with a 237-mL drink containing 250 kcal and 9 g whey protein. Before their operation, participants will fast from solids for 6 h prior to their operation. The staff nurse in charge will monitor the anaesthetic risk of drinking whey-protein-infused CHO drinks and ensure that the participants finish the specific drinks prior to surgery. Participants will be provided with the same specific clear fluid (474 mL which provides 500 kcal and 18 g whey protein) after 4 h post operation (without the presence of bowel sounds) and reviewed by a dietitian. When they can tolerate at least 500 mL specific clear fluids, they are given a regular solid diet.

### Control group

The participants in the control group will fast from 12 midnight on the day of operation with the last meal taken a minimum of 12 h before their operation. On the first post-operative day, participants will be reviewed by a gynaecologist. The group will be allowed clear fluids once there are audible bowel sounds. After having tolerated clear fluids, they will proceed with nourishing fluids and then a soft diet and they will only receive a regular solid diet after tolerating the soft diet.

### Discharge criteria

A gynaecological surgeon will determine the time to discharge of participants according to the discharge criteria. Discharge will be allowed based on pre-established criteria (orally administered pain management, independent mobilisation, sufficient food intake, normal gastrointestinal function resumed, and no suspicion of complications) [13].

### Criteria for withdrawal

The participation will be voluntary, and participants will be free to withdraw from the study at any time without giving any reason and this will in no way affect the participant’s future treatment.

### Adverse events and data safety monitoring

There are no serious side effects identified and a low anaesthetic risk of drinking whey protein 3 h before operation (vomiting/nausea) in this study. If there is any adverse event during the study in any of the participants, it will be recorded in the electronic medical record. If the adverse event is a direct result of the study product or from a medical procedure required for this study, the participant will be referred to a physician as soon as possible and the expenses involve will be paid by the sponsor. There is no additional medication/treatment involved in the study. The staff nurse in charge will monitor the participants if there are any adverse events after consuming the study product. If there are any adverse events/inter-current illnesses, the staff nurse in charge will report to the gynaecological medical officer in charge immediately. The medical officer will initiate appropriate treatment. The study findings will potentially improve treatment outcomes. The expected benefit outweighs the minimal risk to participants and, thus, this study should be supported. If any injuries do occur as a direct result of participating in the study, treatment for such injuries will be provided by the sponsors. There is not pro rata payment for participation in this study.

### Protocol amendment

Any modifications to the protocol which may impact on the conduct of the study, potential benefits for the patient, or may affect patient safety, including changes in study objectives, study design, patient population, sample sizes, study procedures, or significant administrative aspects, will require a formal amendment to the protocol. Such amendments will be agreed upon by the National Cancer Institute and Universiti Putra Malaysia and approved by the Medical Research and Ethics Committee (MREC) prior to implementation and notified to the health authorities in accordance with local regulations.

### Data collection procedure

Data will be collected at baseline, during post-operative hospitalisation and upon discharge post operation. Table 1 illustrates the framework for data collection procedures and the study outcomes.

**Table 1:** Data collection procedures and the study outcomes

Procedure/parameters	Location	Baseline	Pre-operation	Post-operation	Post-operative hospitalisation	Discharge	1 month post discharge
Enrolment	**Clinic**						
Allocation	**Clinic**						
Data collection	**Ward**						
Age		√					
Ethnicity		√					
Education level		√					
Employment status		√					
Marital status		√					
Diagnosis		√					
Other comorbidity		√					
Cancer stage				√			
ASA score		√					
Height		√				√	
Weight changes in the past 1month		√					
Body composition (weight, muscle mass, FM, FFM and MUAC)		√				√	
Handgrip strength		√				√	
PG-SGA		√					
Dietary recall		√			√	√	
Haemoglobin level			√	√			
Glucose level			√	√			
C-reactive protein			√	√			
Albumin			√	√			
Length of post-operative stay						√	
Length of bowel function					√		
Length of solid-food toleraance					√		
Length of clear-fluid tolerance					√		
Post-operative complications (PONV, ileus and infection)					√		
Readmission within 1 month post discharge							√
Reason for readmission (infection, and wound debridement)							√

*ASA* American Society of Anaesthesiologists, *FM* fat mass, *FFM* fat-free mass, *MUAC* mid-upper-arm circumference, *PG-SGA* Patient Generated Subjective Global Assessment, *PONV* post-operative nausea and vomiting

### Outcomes measurement

#### Baseline characteristics

Participant characteristics, such as socio-demographic data (age, ethnicity, education level and marital status), clinical characteristic (diagnosis, other comorbidities and American Society of Anaesthesiologists (ASA) score), nutritional status (Patient Generated Subjective Global Assessment (PG-SGA) score, weight change in the past 1 month, height, body composition and dietary intake), functional status (handgrip strength) and biochemical profile (haemoglobin, C-reactive protein, albumin and glucose levels) will be collected.

#### Primary outcomes

The primary outcome will be the between-group difference in length of post-operative outcomes (length of post-operative hospital stays, clear-fluid tolerance, food tolerance and return of bowel function) between the intervention and control groups. Length of post-operative hospital stay is defined as the time from the operation ending to discharge from the hospital. Length of clear-fluid tolerance is defined as the time from the operation ending to tolerating clear fluids. Length of food tolerance is defined as the time from the operation ending to tolerating regular food. Length of bowel function return is defined as the time from the operation ending to flatus or bowel opening resuming. Primary outcomes will be assessed by a gynaecological surgeon and recorded on a data collection form (progress in ward form) by the nurse in charge.

#### Secondary outcomes

##### Post-operative complications

Post-operative complications including post-operative nausea and vomiting (PONV), ileus and infection, will be monitored and recorded by the surgical gynaecological team. Overall complications were assessed during hospital stay; complication rates were defined per patient; a patient could have suffered from one or several complications [14].

##### Readmission within 1 month post discharge

Readmissions of study participants will be documented from the day of discharge until 1 month (30 days) post-operatively. Readmission complications will be described separately from complications during hospital stay.

#### Other outcomes measures

##### Nutritional status

The changes within group (pre- and post-operation) and between groups (intervention and control group) for body composition (weight and muscle mass) and mid-upper-arm circumference (MUAC) will be assessed. A calibrated TANITA body composition analyser will be used to assess body composition and a calibrated SECA measuring tape for MUAC.

### Biochemical profile

The changes within group (pre- and post-operation) and between groups (intervention and control group) for biochemical profile (haemoglobin, C-reactive protein, albumin and glucose levels) will be measured. The results of biochemical profiles of the study participants will be accessed from the medical record system.

### Functional status

The changes within group (pre- and post-operation) and between groups (intervention and control group) for functional status (handgrip strength) will be measured by using a calibrated JAMAR® hand dynamometer. Scores of three successive trials for non-dominant hand-testing. will be recorded The average score of the three trials will be used to interpret grip-strength performance.

### Confidentiality, handling and storage of data documents

Participants’ names will be kept anonymous and will be linked only with a study identification number for this research for confidentially purposes. The identification number instead of patient identifiers will be used on participant data sheets. Digital documents and data will be kept in password-protected applications and folders. The hardcopy documents will be stored in the investigators locked office and maintained for a minimum of 5 years after study completion. Participants will not be allowed to view their personal study data, as the data will be consolidated into a database.

### Sample size calculation

The study will be powered to detect differences between the intervention and control groups in the primary outcomes (post-operative outcomes). Sample size of the study is calculated by using a formula which was proposed by Woodword [25]. According to the results of a previous study by Balayla et al. [20], the study needs about 33 participants per group. After adjustment for an 80% response rate and a 90% expected eligible rate (power = 80%, alpha level = 0.05), the study plans to recruit a total of 110 participants (55 participants each group) to account for a 20% drop-out rate.

### Statistical analysis

All randomised RCT participants will be included in the analysis on an intention-to-treat (ITT) basis. The ITT analysis includes every participant who is randomised according to the randomised treatment assignment. According to Gupta [26], the ITT analysis reflects the practical clinical scenario because it admits noncompliance and protocol deviations, maintains the prognostic balance generated from the original random treatment allocation as well as giving an unbiased estimate of treatment effect.

The analyses will be performed using IBM SPSS (version 23.0). Descriptive statistics will be utilised for the described participants’ characteristics. Data normality will be checked by using the Shapiro-Wilk test and reconfirmed by visual inspection of a histogram and stem-leaf plots. Numerical data will be presented as mean ± standard deviation or median (interquartile range) as appropriate with categorical data as frequency and percentage.

A comparison of numerical data, which is normally distributed between the two groups, will be analysed using the independent samples’ *t* test while the Mann-Whitney *U* test will be used if not normally distributed. Pearson’s chi-square test will be used to study the association between categorical data and categorical data while Fisher’s exact test will be used if the assumptions of Pearson’s chi-square test for independence are not met. A multi-linear regression test will be used to determine factors related to POHS among surgical GC patients. All probability values will be used two-sided and a level of significance of less than 0.05 (*p* value < 0.05) will be considered as statistically significant [27].

### Dissemination

This study will be conducted according to the principles of the latest Declaration of Helsinki, the Medical Research Involving Human Subjects Act (WMO) and the Good Clinical Practice standards (GCP). The study is investigator-initiated. We used the Standard Protocol Items: Recommendations for Interventional Trials (SPIRIT) Checklist when writing this report [28].

## Discussion

The FTR surgery programme in Malaysia has mostly been conducted in gastroenterology (upper gastrointestinal and lower gastrointestinal) surgery and even in hepato-biliary surgery [29]. All of the post-operative outcomes in FTR studies are very convincing whereby they show shortened LOS, faster resumption of food tolerance and lower readmission rates. Although the impact of FTR on nutritional status, anti-inflammatory effects and functional status among surgical gastric cancer patients has been published by Makuuchi et al. [30], the impact of FTR among surgical GC patients remain unknown. The effectiveness of FTR in surgical GC patients also remains unexplored, especially in Malaysia.

Most of the available FTR surgery studies [7–10] have investigated the effectiveness of a solely CHO-containing solution as the CHO-loading formula. An available study by Perrone et al. [11] showed a positive impact of whey-protein CHO-loading among cholecystectomy patients. However, the formula containing whey protein infused with a CHO drink has not been widely implemented as the CHO-loading drink among surgical GC patients. To our best knowledge, this is the first study that used a formula containing whey-protein infused with a CHO drink as the CHO-loading drink among surgical GC patients in Malaysia.

The value of pre-operative overnight fasting has come under doubt recently as, in fact, the incidence of surgery-related aspiration is very low and clinically important consequences of aspiration are even rarer [31]. Moreover, the appropriateness of overnight fasting has been raised because using it CHO reserves will become depleted and cause changes in the metabolism and metabolic responses [32]. Both nutritional compromise going into surgery and a prolonged pre-operative overnight fasting state worsen glycogen and muscle-mass depletion [33].

After a major operation, conventional post-operative care usually includes restrict oral intake to prevent post-operative ileus and, thus, to protect surgical anastomoses [34]. The other reason for nil-by-mouth post operation is to allow anastomoses time to heal before being stressed by the food transiting the gut [35]. In fact, 1–2 L of fluid is secreted by the stomach and pancreas and then absorbed by the small intestine daily [36, 37]. Hence, patients actually tolerated a high volume of fluid [38]. Moreover, within 24 h, starvation changes the body’s metabolism by increasing insulin resistance and depleting muscle function [39]. Early oral feeding post-operatively has been demonstrated to hasten the return of bowel function, improve oral tolerance and shorter LOS [21, 40]. Based on these study findings, nil-by-mouth post-operatively does not seem to be reasonable or beneficial.

Thus, we hypothesise that the FTR programme with a whey-protein-infused CHO drink as the CHO-loading drink pre-operatively, and early oral feeding post-operatively, can result in more positive post-operative outcomes including shortened LOS, catabolic minimisation in order to preserve muscle mass or functional status, and anabolic promotion among surgical GC patients. In the Malaysia healthcare system, this may lead to an immense advantage that may be achieved by the accomplished execution of an FTR surgery programme at an institutional level.

## Trial status

This study protocol version 3, dated 27 September 2017, received ethical approval by the MREC, Ministry of Health, Malaysia on 27 September 2017. The protocol was retrospectively registered at ClinicalTrials.gov on 12 September 2018. Data recruitment started on 3 October 2017 and completed on 27 September 2019. This study protocol was submitted for publication on 21 May 2019 while the recruitment was ongoing and before the last patient’s last visit.

## Supplementary information

**Additional file 1.** Participant information sheet & consent form.

## Data Availability

The current database/materials for the study are available from the corresponding author on reasonable request.

## Abbreviations

ASA: American Society of Anaesthesiologists
CHO: Carbohydrate
CHO-P: Intervention group
CO: Control group
CONSORT: Consolidated Standards of Reporting Trials
ERAS: Enhanced Recovery After Surgery
FTR: Fast-track-recovery
GC: Gynaecological cancer
ITT: Intention-to-treat
LOS: Length of hospital stay
MREC: Medical Research Ethics Committee
MUAC: Mid-upper-arm circumference
PONV: Post-operative nausea and vomiting
SPIRIT: Standard Protocol Items: Recommendations for Interventional Trials
RCT: Randomised controlled trial
