# Supplementary material for: Fast-track- recovery surgery with a whey-protein-infused carbohydrate-loading drink pre-operatively and early oral feeding post-operatively among surgical gynaecological cancer patients: study protocol of an open-labelled, randomised controlled trial
Source: Trials. 2020 Jun 16;21:533. doi: 10.1186/s13063-020-04462-4 (PMC7298941; doi:10.1186/s13063-020-04462-4)
Supplement: Supplementary file 1 — Additional file 1. Participant information sheet & consent form. [file 13063_2020_4462_MOESM1_ESM.docx]

Additional file

**Participant Information Sheet and Informed Consent Form**

(for adult subjects and intervention studies)

**1. Title of study:**

Fast Track Recovery Surgery with A Whey Protein Infused Carbohydrate Loading Drink Pre-Operatively and Early Oral Feeding Post-Operatively among Surgical Gynaecologic Cancer Patients

**2. Name of investigator and institution:**

**Name and Institution of Principal investigator:**

Ho Chiou Yi, Dietitian at National Cancer Institute, Putrajaya,

Postgraduate research student at Universiti Putra Malaysia

**Name and Institution of Co-Investigators:**

Dr. Zuriati Binti Ibrahim, Senior lecturer and dietitian Universiti Putra Malaysia

Dr. Zalina Binti Abu Zaid, Senior lecturer Universiti Putra Malaysia

Dr. Zulfitri 'Azuan Bin Mat Daud, Senior lecturer Universiti Putra Malaysia

Dr. Nor Baizura Binti Md Yusop, Senior lecturer Universiti Putra Malaysia

**3. Introduction:**

This study is an open labelled randomized trial study. There is experimental intervention being administered.

We would like to invite you to take part in our research study because you have gynaecology cancer. Before you decide we would like you to understand why the research is being done and what it would involve for you. One of our team members will go through the information sheet with you and answer any questions you have. We’d suggest this should take about 5-10 minutes. Ask us if there is anything that is not clear. After you are properly satisfied that you understand this study, and that you wish to participate, you must sign this informed consent form.

Your participation in this study is voluntary. You do not have to be in this study if you do not want to. You may also refuse to answer any questions you do not want to answer. If you volunteer to be in this study, you may withdraw from it at any time. If you withdraw, any data collected from you up to your withdrawal will still be used for the study. Your refusal to participate or withdrawal will not affect any medical or health benefits to which you are otherwise entitled. There is no prorated payment for participation in this study.

A total of 110 subjects from multidiscipline outpatient clinic in National Cancer Institute, Putrajaya will be participating in this study. The whole study will last about 8 months. The participation duration for each subject is minimum 1 week until patient is discharged. Probability for randomization into group A and group B are the same. If you agree to participate in the study, you will be assigned into either group A or B.

The product used in this study does not contain porcine and bovine ingredient and has been certified HALAL by Singapore Authorities. This study has been approved by the Medical Research and Ethics Committee, Ministry of Health Malaysia.

**4. What is the purpose of the study?**

This study is to determine the impact of fast track recovery feeding with a whey protein plus carbohydrate drink among surgical gynaecologic oncology patients in National Cancer Institute

**5. What will happen if I decide to take part?**

1. In this study, we will collect information on your nutritional status, physical function and hospitalization record.
2. At first, we will collect information about your medical condition from your medical record at the time of follow up in medical outpatient clinic, including your blood laboratory investigations. All blood laboratory investigations are extracted from your medical outpatient record and not taken specific to the purpose of this study. This trial does not involve collecting biological specimens for storage. There will be no biological specimens collected for genetic or molecular analysis in this trial and future use in ancillary studies.
3. A dietician will assess your nutritional status in diet clinic. Your nutritional status will be assessed using a questionnaire; dietary recall and measurements of your height, weight, body fat percentage and handgrips at the arm will be assessed. Weight, height and body fat percentage will be measured using a special scale that requires you to stand on the scale.
4. Upon admission, you have to attend to diet clinic to assess of your height, weight, body fat percentage and handgrips.

- If you are assigned to group A, you need to be fasting at 12 midnight and only allowed to take water till 3 hours before operation.
- If you are assigned to group B, you are allowed to take solid food as hospital served 6 hours before operation. 3 hours before operation, you will be provided 1 pack supplement (a lactose-free clear tea-colour fruit flavoured fluid contain 14% whey protein, 86% carbohydrates and 0% lipids).

Upon discharge from ward, you have to attend to diet clinic to assess of your weight, body fat percentage and handgrips.

1. After completion of study, continuous treatment (diet intervention & oncological treatment) will be given as routine care. Study product will not be continued. But high protein nourishing fluid will only be given if indicated (malnutrition) after dietary assessment.

**6. What are my responsibilities when taking part in this study?**

It is important that you answer all of the questions asked by the study staff honestly and completely as well as compliance to diet advice and oral supplement as planned.

**7. What are the potential risks and side effects of being in this study?**

You may feel slight tired during the measurement of handgrip strength at the arm, which will resolve spontaneously after the measurement is completed. During admission to ward, branula will be set as routine care & which will cause discomfort.

Others potential risk and side effects of being in this study (group B) is intolerance towards whey protein plus carbohydrate drink provided such as nausea, diarrhoea or vomiting. If such intolerance occurs by the subjects, these steps will be taken into measure:

- To substitute with carbohydrate drink only and to ask subjects to withdraw from the study
- To stop the prescription of whey protein plus carbohydrate drink and to ask subjects to withdraw from the study
- Referral to doctor if necessary

**8. What are the benefits of being in this study?**

There may or may not be any benefits to you. This study does provide a better understanding of the disease/condition studied. It may provide benefit for surgical gynecologic oncology patients in the future in terms of development of new dietary intervention protocol in cancer patient ongoing for surgery treatment.

**9. What if I am injured during this study?**

If you are injured as a result of being in this study, you should contact your investigator. In the event of a bodily injury or illness directly resulting from the study product or a medical procedure required for this study which is very minimal, you will be referred to a medical doctor as soon as possible and the expenses involve will be paid by the sponsor. The sponsor is not responsible for medical expenses due to pre-existing medical conditions, any underlying disease, any ongoing treatment process, your negligence or wilful misconduct, the negligence or wilful misconduct of your investigator or the study site or any third parties. You do not lose any of your legal rights to seek compensation by signing this form.

**10. What are my alternatives if I do not participate in this study?**

You do not have to participate in this study to get treatment for your disease or condition. Alternative treatments of routine care in dietary interventions post-surgery are available in Institut Kanser Negara and most of government hospitals. You always can get diet intervention post-surgery and usage of high protein nourishing fluid will be depending on your daily energy and protein requirement to support post-surgery wound healing and optimize nutritional status.

**11. Who is funding this research?**

There is no funding involved in this research.

**12. Can the research or my participation be terminated early?**

The research officer may due to concerns for your safety, stop the study or participation at any time. If the study is stopped early for any reason you will be informed and arrangements made for your future care. You may be asked to attend a final follow up visit.

**13. Will my medical information be kept private?**

All your information obtained in this study will be kept and handled in a confidential manner, in accordance with applicable laws and/or regulations. Principal investigator and co-investigator in this study only are allowed to assess to medical records and data. The identification number instead of patient identifiers will be used on subject data sheets. All data will be entered into a computer that is password protected. When publishing or presenting the results, your identity will not be revealed without your expressed consent. Individuals involved in this study and in your medical care, and governmental or regulatory authorities may inspect and copy your medical records, where appropriate and necessary. With your permission, your family will be informed that ypur participation in this study. You will not be informed of study findings. You will be informed if new information becomes available relevant to consent. All data of study might be sent to overseas for analysis but your identity will not be exposed all the time.

**14. Who should I call if I have questions?**

If you have any questions about the study or you think you have a study related injury or you want information about treatment, please contact the researcher as below:

Ho Chiou Yi

Dietitian U 41, Dietetic and Food Service Department, National Cancer Institute

03-88923406

dtho@nci.gov.my

If you have any questions about your rights as a participant in this study, please contact: The Secretary, Medical Research & Ethics Committee, Ministry of Health Malaysia, at telephone number 03-2287 4032.

**INFORMED CONSENT FORM**

Title of Study: Fast Track Recovery Surgery with A Whey Protein Infused Carbohydrate Loading Drink Pre-Operatively and Early Oral Feeding Post-Operatively among Surgical Gynaecologic Cancer Patients: Study Protocol of an Open Labelled Randomized Controlled Trial

By signing below I confirm the following:

- I have been given oral and written information for the above study and have read and understood the information given.
- I have had sufficient time to consider participation in the study and have had the opportunity to ask questions and all my questions have been answered satisfactorily.
- I understand that my participation is voluntary and I can at any time free to withdraw from the study without giving a reason and this will in no way affect my future treatment. I understand the risks and benefits, and I freely give my informed consent to participate under the conditions stated. I understand that I must follow the study doctor’s instructions related to my participation in the study.
- I understand that study staff and government or regulatory authorities have direct access to my medical record in order to make sure that the study is conducted correctly and the data are recorded correctly and the data are recorded correctly. All personal details will be treated as STRICTLY CONFIDENTIAL.
- I will receive a copy of this subject information/ informed consent form signed and dated to bring home.

**Subject:**

Signature: I/C number:

Name: Date:

**Investigator conducting informed consent:**

Signature: I/C number:

Name: Date:

**Impartial witness:**

Signature: I/C number:

Name: Date:
